# The relationship between the CUN-BAE body fatness index and incident diabetes: a longitudinal retrospective study

**DOI:** 10.1186/s12944-023-01784-5

**Published:** 2023-02-06

**Authors:** Qing Peng, Zihao Feng, Zhuojian Cai, Dixing Liu, Jiana Zhong, Hejia Zhao, Xiuwei Zhang, Weikun Chen

**Affiliations:** 1 Second Clinical Medical College, Southern Medical University, Guangzhou, China; 2 First Clinical Medical College, Southern Medical University, Guangzhou, China; 3 Department of Endocrinology, Affiliated Dongguan Hospital, Southern Medical University (Dongguan People’s Hospital), No. 3, South Wandao Road, Xingu Chong, Wanjiang District, Dongguan, 523059 Guangdong Province China; 4 Department of Endocrinology, The Dongguan Affiliated Hospital of Jinan University, Binhaiwan Central Hospital of Dongguan, Dongguan, China; 5 School of Public Health, Southern Medical University, Guangzhou, China

**Keywords:** CUN-BAE index, Obesity, Type 2 diabetes mellitus, Body fatness, Insulin resistance

## Abstract

**Background:**

The Clínica Universidad de Navarra-Body Adiposity Estimator (CUN-BAE) index has been recommended as an ideal indicator of body fat and exhibited significant correlation with cardiometabolic risk factors. However, whether the CUN-BAE index correlates with incident diabetes in Asian populations is unknown. Therefore, this longitudinal study was designed to evaluate the association between baseline CUN-BAE index and type 2 diabetes mellitus (T2DM).

**Methods:**

This retrospective longitudinal study involved 15,464 participants of 18–79 years of age in the NAGALA (NAfld in the Gifu Area Longitudinal Analysis) study over the period of 2004–2015. Cox proportional hazards regression was performed to test the relationship between the baseline CUN-BAE index and diabetes incidence. Further stratification analysis was conducted to ensure that the results were robust. The diagnostic utility of the CUN-BAE index was tested by the receiver operating characteristic (ROC) curve.

**Results:**

Over the course of an average follow-up of 5.4 years, 373 (2.41%) participants developed diabetes. A higher diabetes incidence was associated with higher CUN-BAE quartiles (*P* for trend< 0.001). Each 1 unit increase in CUN-BAE index was associated with a 1.08-fold and 1.14-fold increased risk of diabetes after adjustment for confounders in males and females, respectively (both *P* < 0.001). Stratification analysis demonstrated a consistent positive correlation between baseline CUN-BAE and diabetes incidence. Moreover, based on ROC analysis, CUN-BAE exhibited a better capacity for diabetes prediction than both body mass index (BMI) and waist circumference (WC) in both sexes.

**Conclusions:**

The baseline CUN-BAE level was independently related to the incidence of diabetes. Increased adiposity determined by CUN-BAE could be used as a strong nonlaboratory predictor of incident diabetes in clinical practice.

## Background

Diabetes is a significant contributor to health-system costs and a significant cause of death and morbidity worldwide [1]. The 10th edition of the IDF Diabetes Atlas reported that by 2045, the prevalence of diabetes is estimated to rise from 10.5% (536.6 million people) in 2021 to 12.2% (783.2 million people) globally [2]. Additionally, its prevalence in Asian regions is notably increasing, and more than 60% of all cases of diabetes occur in these regions [3, 4]. The risk of diabetic neuropathy, cerebrovascular disease, and even carcinoma is significantly increased in patients with diabetes [3, 5–8]. Therefore, there is an urgent need for efficient and simple methods to improve early detection, especially in Asian populations.

Obesity, which refers to an overabundance of body fat, is a recognized risk factor for diabetes [9, 10]. Although BMI is a traditional diagnostic method that is most frequently employed in the present classification system, its ability to discriminate between lean and fat mass is limited and varies by sex and age [11, 12]. In addition, the waist-to-hip ratio or waist circumference is used to reflect the degree of abdominal fat accumulation, but they are insufficient for a comprehensive assessment of body fat mass [13, 14]. Body fat percentage (BF%) has been proven to be an effective and robust method for identifying obesity and the risk of obesity-related diabetes [15, 16]. It has been recognized that extra body fat could result in insulin resistance and thus promote the occurrence and development of diabetes [17–20]. Furthermore, BF% has been confirmed as a risk factor that affects cardiometabolic function independently of BMI and abdominal obesity in previous studies [13, 21, 22]. Thus, BF% may be a notable index for predicting individuals at high risk of obesity-related type 2 diabetes.

Currently, various methods, including magnetic resonance imaging (MRI) and dual-energy X-ray absorptiometry (DXA), can be used for the measurement of BF% [11]. However, their wide application is largely limited by high costs and radiation exposure [23, 24]. Recently, a newly developed alternative anthropometric method, the CUN-BAE index, has attracted much attention. This BF% estimator is based on the age, sex and BMI of Caucasian subjects [25]. A strong association between CUN-BAE and BF% was identified in a previous study, and this association was stronger than that of other anthropometric measurements [25, 26]. In addition, the CUN-BAE index was better than BMI or WC at predicting cardiometabolic risk factors [25, 27–29]. In this regard, it is already known that CUN-BAE is an optimal and accessible method for estimating BF% and identifying people at high risk for metabolic disorders. However, the CUN-BAE index has not been extensively studied for the prediction or identification of diabetes to the best of our knowledge [28]. Thus, CUN-BAE was evaluated for its importance and contribution to the prediction of diabetes in adulthood in Asian individuals in the current study.

## Methods

### Data source

The Dryad Digital Repository (www.datadryad.org) provided the original data, which were openly published and freely available to researchers. The Dryad database was created in September 2008 and funded by the US National Science Foundation. This database was used to deposit high-quality data resources, making the data underlying scientific publications freely reusable, discoverable and citable. In the current study, these population-based longitudinal data were available from this online database, which was originally analysed and released by Professor Okamura [30]. The original data contained the following variables: age, sex, waist circumference, fatty liver, fasting plasma glucose (FPG), body mass index (BMI), exercise habit, smoking status, glycosylated haemoglobin (HbA1c), and diastolic and systolic blood pressure (DBP and SBP). In addition, gamma-glutamyl transferase (GGT), lipid profile (total cholesterol, triglycerides, and HDL-C) alanine aminotransferase (ALT), aspartate aminotransferase (AST) and diabetes incidence was also included in this database. Furthermore, each of the participants was required to complete a survey that included lifestyle variables, such as alcohol consumption, physical activities and smoking status. Three groups of individuals were categorized based on their smoking habits: nonsmokers, former smokers, and current smokers. Drinking habits were divided into high consumption (> 280 g/week), moderate consumption (140–280 g/week), light consumption (40–140 g/week), and minimal consumption (< 40 g/week). For physical exercise, patients who worked out more than once a week were characterized as standard exercisers.

### Study population

**Fig. 1:**
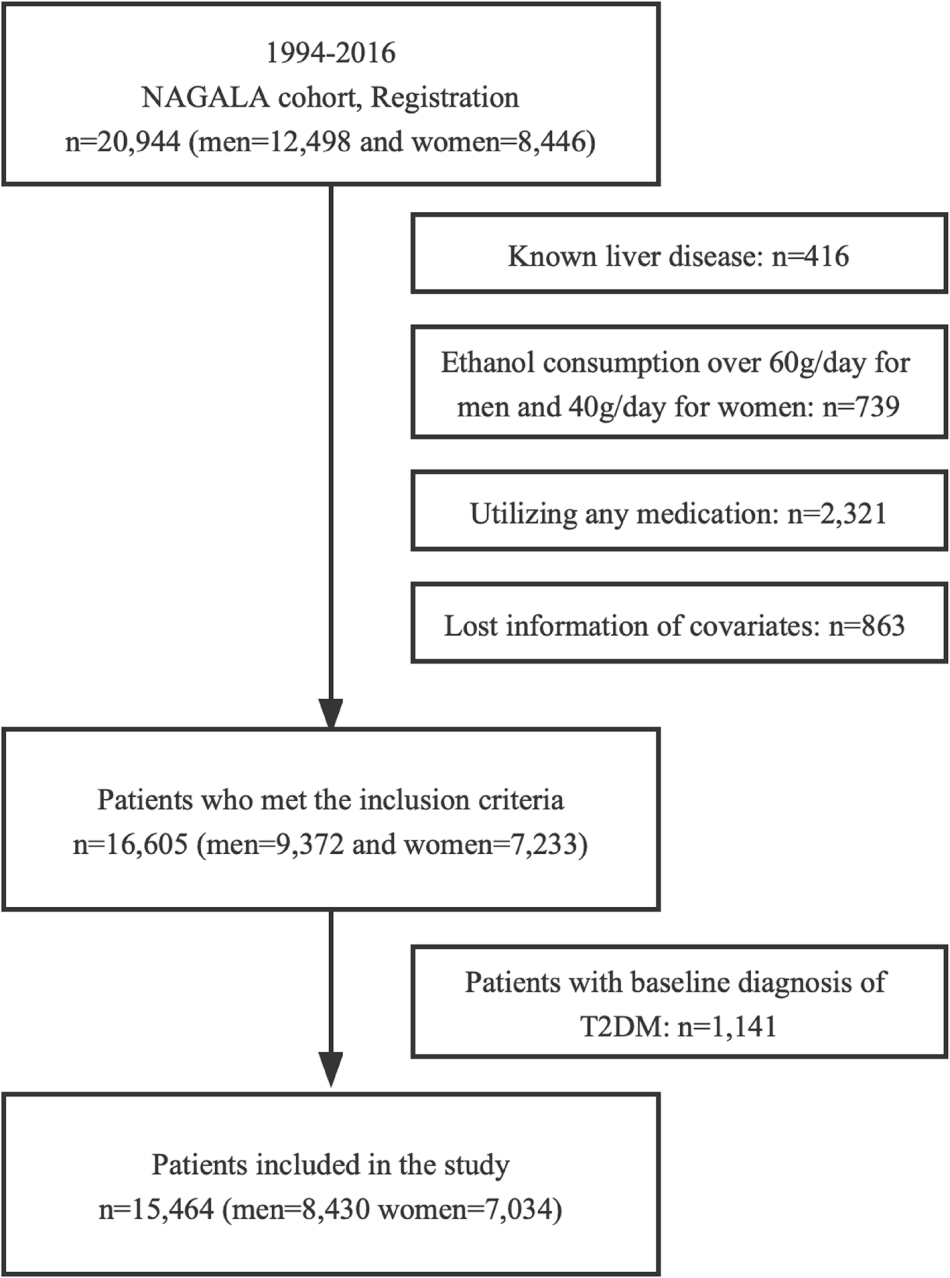
Flowchart of patient enrollment. Abbreviations: T2DM, type 2 diabetes mellitus

Study participants included all patients who participated in Murakami Memorial Hospital’s physical examination program between 2004 and 2015. In addition, an incident diabetes follow-up study was conducted. These participants were enrolled over different time points, and most of them received exams annually. In this study, the median follow-up time was 5.4 years (1967 days), ranging from 0.5 years (164 days) to 13.0 years (4732 days). Individuals who met one of the following criteria were excluded: (1) alcoholic fatty liver disease; (2) diabetes at baseline; (3) utilization of any medication; (4) hepatitis B antigen and hepatitis C antibody found at baseline in patients with viral hepatitis; and (5) lost information on covariates. The study ultimately included 15,464 subjects, 7034 of whom were females and 8430 of whom were males (Fig. 1).

### Measurement of the CUN-BAE index

CUN-BAE = − 44.988 + (3.172 × BMI) + (10.689 × sex) + (0.503 × age) – (0.026 × BMI^2^) + (0.181 × BMI × sex) – (0.02 × BMI × age) + (0.00021 × BMI^2^ × age) – (0.005 × BMI^2^ × sex).

Male = 0; Female = 1.

### Ascertainment of diabetes

The criteria for diagnosing diabetes were HbA1c no less than 6.5%, fasting plasma glucose no less than 7 mmol/L, or reporting diabetes diagnoses on the follow-up questionnaire.

### Statistical analysis

We stratified baseline characteristics based on sex because males and females have significantly different body compositions. Then, four groups were formed according to CUN-BAE quartiles (Q1-Q4). Continuous variables are presented as means and standard deviation. Categorical data are presented as frequencies and percentages. For variables with a normal distribution, one-way ANOVA was performed, and variables with nonnormal distributions were tested using the Kruskal–Wallis test. Categorical variables among groups were compared using the chi-square test. Univariate and multivariate Cox regression analyses were applied to calculate the hazards ratio (HR) and 95% confidence intervals (CIs) between the baseline CUN-BAE index and diabetes after adjusting for age, BMI (category), smoking status, alcohol consumption, fatty liver disease, GGT, HDL, TGs, TC, SBP and exercise habits. These may be important factors in T2DM development as a previous study has shown [31]. BMI (category) was calculated according to WHO criteria for Asian individuals (< 24 kg/m^2^; ≥24 kg/m^2^ and < 28 kg/m^2^; ≥28 kg/m^2^) [32]. Then, the differences among different CUN-BAE quartile groups were determined by log-rank tests.

Furthermore, ROC curves were also drawn to measure the diagnostic efficacy of different anthropometric parameters. The results were further validated through stratification analysis and likelihood ratio tests according to the following factors: sex, BMI, age, SBP, fatty liver disease, HDL-C, GGT, TC, TGs, smoking status, exercise habits and alcohol consumption. Statistical analysis and data processing were carried out in R (http://project.r-project.org) and Empower Stats (http://www.empowerststs.com, X&Y Solutions, Inc., Boston, MA). A probability of *P <* 0.05 was used as a measure of statistical significance (two-sided).

## Results

### Study population characteristics

**Fig. 2:**
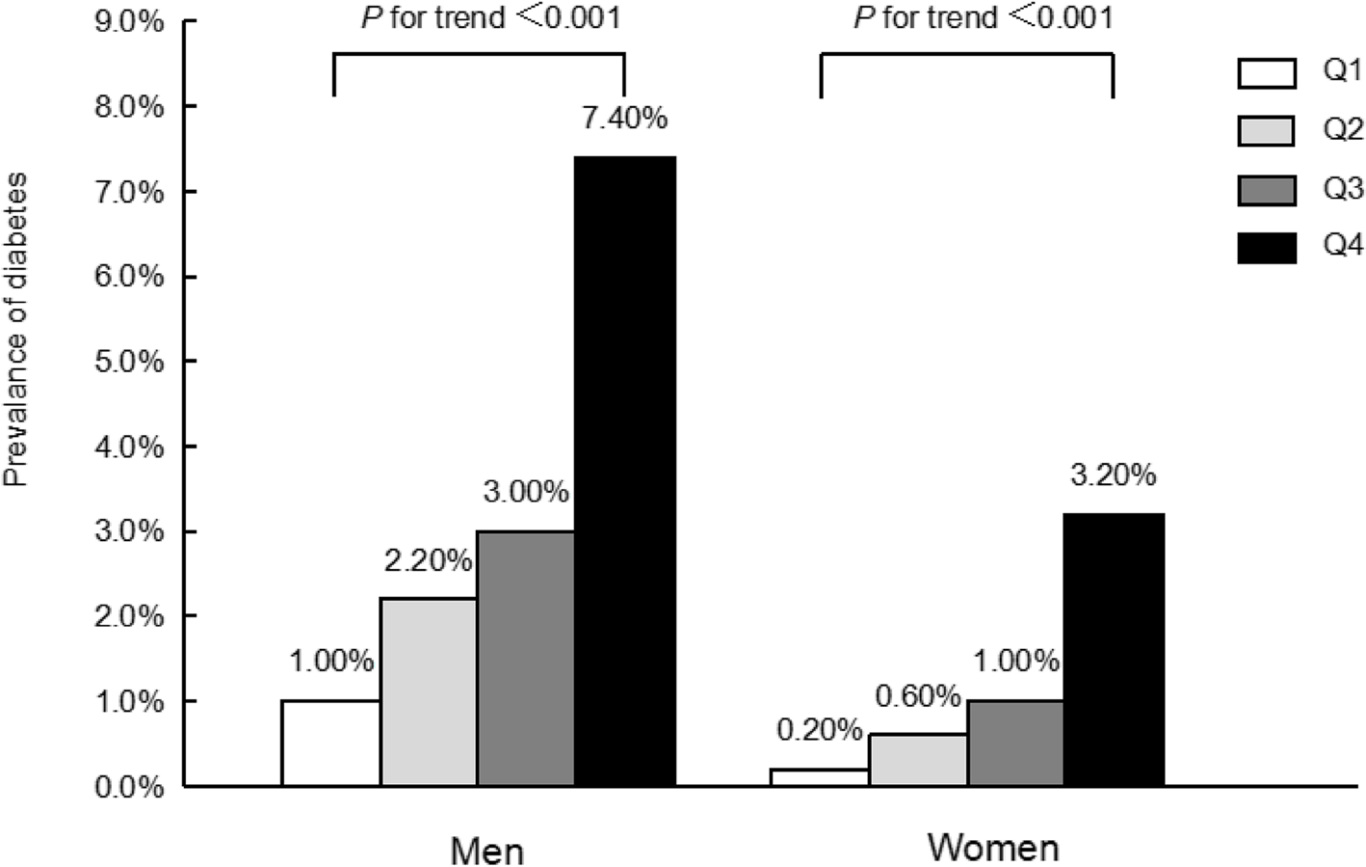
Prevalence of diabetes according to the baseline CUN-BAE index quartiles. Notes: All of the study participants were divided into four groups according to quartiles of CUN-BAE index (In male: quartile 1 [Q1]: < 17.826; quartile 2 [Q2]: 17.826 to ≤20.862; quartile 3 [Q3]: 20.868 to ≤23.945; quartile 4 [Q4]: > 23.945. In female: quartile 1 [Q1]: < 26.374; quartile 2 [Q2]: 26.374 to ≤29.434; quartile 3 [Q3]: 29.434 to ≤32.833; quartile 4 [Q4]: > 32.833). The prevalence of diabetes increased with ascending quartiles of CUN-BAE index (*P* for trend < 0.05). **A** Males; **B** Females

Approximately 15,464 participants without diabetes participated in the present study (average age of 43.7 ± 8.9 years). Overall, 8430 males (54.5%) and 7034 females (45.5%) were included in this study. Males and females demonstrated significant differences in anthropometric indicators, blood pleasure values, lifestyles and laboratory indicators. The average CUN-BAE index in females was remarkably higher than that in males (29.8 ± 4.9 vs. 20.9 ± 4.7). As demonstrated in Table 1, participants in the top quartile of the CUN-BAE (Q4) presented with older age, higher waist circumference, higher BMI, higher levels of AST, ALT, GGT, FBG, DBP, SBP, TC, TGs, and HbA1c, a higher incidence of fatty liver and a higher percentage of smokers and drinkers compared with the other groups (Q1-Q3) (all *P values* < 0.001). In addition, it was remarkable that in both males and females, diabetes incidence increased with increasing quartiles of the baseline CUN-BAE index (*P* for trend< 0.001) (Fig. 2).

**Table 1:** Baseline characteristics of study participants by gender according to CUN-BAE index quartiles

	Males				*P* value	Females				*P* value
	Q1(< 17.826)	Q2(17.826 to ≤20.862)	Q3(20.862 to ≤23.945)	Q4(> 23.945)		Q1(< 26.374)	Q2(26.374 to ≤29.434)	Q3(29.434 to ≤32.833)	Q4(> 32.833)	
Total (n)	2108	2107	2107	2108		1759	1758	1758	1759	
Age (years)	39.7 ± 7.4	43.5 ± 8.2	46.2 ± 8.9	47.0 ± 9.4	< 0.001	36.9 ± 6.9	41.8 ± 7.1	45.8 ± 7.9	48.5 ± 8.4	< 0.001
BMI (kg/m^2^)	19.8 ± 1.3	22.0 ± 1.0	23.7 ± 1.0	26.8 ± 2.3	< 0.001	18.1 ± 1.1	19.8 ± 0.9	21.3 ± 1.1	24.8 ± 2.6	< 0.001
WC (cm)	72.2 ± 4.5	78.0 ± 4.1	82.3 ± 4.2	89.3 ± 6.3	< 0.001	64.9 ± 4.4	68.8 ± 4.8	72.5 ± 5.3	80.5 ± 7.6	< 0.001
Weight (kg)	58.3 ± 5.7	64.5 ± 5.7	68.7 ± 6.2	77.7 ± 9.7	< 0.001	46.0 ± 4.1	50.1 ± 4.3	53.4 ± 4.8	61.2 ± 8.1	< 0.001
SBP (mmHg)	111.9 ± 12.0	116.6 ± 12.5	120.1 ± 13.4	126.5 ± 14.3	< 0.001	102.8 ± 11.2	106.2 ± 12.6	110.1 ± 13.0	118.4 ± 15.2	< 0.001
DBP (mmHg)	69.7 ± 8.4	73.4 ± 9.1	76.1 ± 9.4	80.3 ± 9.7	< 0.001	63.5 ± 7.7	65.6 ± 8.9	68.0 ± 9.2	73.4 ± 10.1	< 0.001
ALT (IU/L)	18.6 ± 8.8	21.4 ± 10.7	24.4 ± 12.7	32.1 ± 19.8	< 0.001	13.7 ± 6.7	14.3 ± 20.9	14.6 ± 6.1	17.6 ± 9.2	< 0.001
AST (IU/L)	18.1 ± 6.9	18.7 ± 7.1	19.6 ± 7.3	22.7 ± 9.9	< 0.001	16.1 ± 5.3	16.5 ± 14.7	16.5 ± 5.5	17.8 ± 6.9	< 0.001
GGT (IU/L)	19.9 ± 17.4	23.6 ± 18.6	28.0 ± 24.7	32.0 ± 23.4	< 0.001	12.3 ± 6.2	12.8 ± 7.0	13.6 ± 10.0	15.9 ± 10.5	< 0.001
FPG (mmol/L)	5.2 ± 0.4	5.3 ± 0.4	5.3 ± 0.4	5.4 ± 0.3	< 0.001	4.9 ± 0.4	4.9 ± 0.4	5.0 ± 0.4	5.2 ± 0.4	< 0.001
HbA1c (%)	5.1 ± 0.3	5.1 ± 0.3	5.2 ± 0.3	5.3 ± 0.3	< 0.001	5.1 ± 0.3	5.1 ± 0.3	5.2 ± 0.3	5.3 ± 0.3	< 0.001
TC (mmol/L)	4.9 ± 0.8	5.1 ± 0.8	5.2 ± 0.8	5.4 ± 0.9	< 0.001	4.7 ± 0.8	5.0 ± 0.8	5.2 ± 0.9	5.4 ± 0.9	< 0.001
Triglycerides (mmol/L)	0.8 ± 0.5	1.0 ± 0.7	1.2 ± 0.8	1.4 ± 0.9	< 0.001	0.5 ± 0.2	0.6 ± 0.3	0.7 ± 0.4	0.9 ± 0.5	< 0.001
HDL-C (mmol/L)	1.5 ± 0.4	1.3 ± 0.4	1.3 ± 0.3	1.2 ± 0.3	< 0.001	1.7 ± 0.4	1.7 ± 0.4	1.6 ± 0.4	1.5 ± 0.4	< 0.001
Smoking status (n, %)					< 0.001					0.195
Never	824 (39.1%)	730 (34.6%)	681 (32.3%)	657 (31.2%)		1533 (87.2%)	1532 (87.1%)	1533 (87.2%)	1541 (87.6%)	
Past	497 (23.6%)	606 (28.8%)	706 (33.5%)	702 (33.3%)		110 (6.3%)	111 (6.3%)	127 (7.2%)	93 (5.3%)	
Current	787 (37.3%)	771 (36.6%)	720 (34.2%)	749 (35.5%)		116 (6.6%)	115 (6.5%)	98 (5.6%)	125 (7.1%)	
Alcohol consumption (n, %)					< 0.001					0.024
No or minimal	1425 (67.6%)	1317 (62.5%)	1269 (60.2%)	1343 (63.7%)		1630 (92.7%)	1595 (90.7%)	1591 (90.5%)	1635 (93.0%)	
Light	328 (15.6%)	375 (17.8%)	354 (16.8%)	312 (14.8%)		92 (5.2%)	104 (5.9%)	106 (6.0%)	87 (4.9%)	
Moderate	251 (11.9%)	287 (13.6%)	326 (15.5%)	302 (14.3%)		37 (2.1%)	59 (3.4%)	61 (3.5%)	37 (2.1%)	
Heavy	104 (4.9%)	128 (6.1%)	158 (7.5%)	151 (7.2%)						
Habit of exercise (n, %)					0.381					< 0.001
No	1756 (83.3%)	1650 (78.3%)	1694 (80.4%)	1730 (82.1%)		1537 (87.4%)	1464 (83.3%)	1441 (82.0%)	1483 (84.3%)	
Yes	352 (16.7%)	457 (21.7%)	413 (19.6%)	378 (17.9%)		222 (12.6%)	294 (16.7%)	317 (18.0%)	276 (15.7%)	
Fatty liver disease (n, %)					< 0.001					< 0.001
No	2016 (95.6%)	1784 (84.7%)	1495 (71.0%)	880 (41.7%)		1758 (99.9%)	1738 (98.9%)	1700 (96.7%)	1352 (76.9%)	
Yes	92 (4.4%)	323 (15.3%)	612 (29.0%)	1228 (58.3%)		1 (0.1%)	20 (1.1%)	58 (3.3%)	407 (23.1%)	

*Abbreviations: BMI* body mass index, *WC* waist circumference, *SBP* systolic blood pressure, *DBP* diastolic blood pressure, *HDL-C* high-density lipoprotein-cholesterol, *FPG* fasting plasma glucose, *HbA1c* glycosylated haemoglobin A1c, *ALT* alanine aminotransferase, *AST* aspartate aminotransferase, *GGT* gamma-glutamyl transferase

### Clinical outcome and Kaplan–Meier analysis stratified by CUN-BAE index

**Fig. 3:**
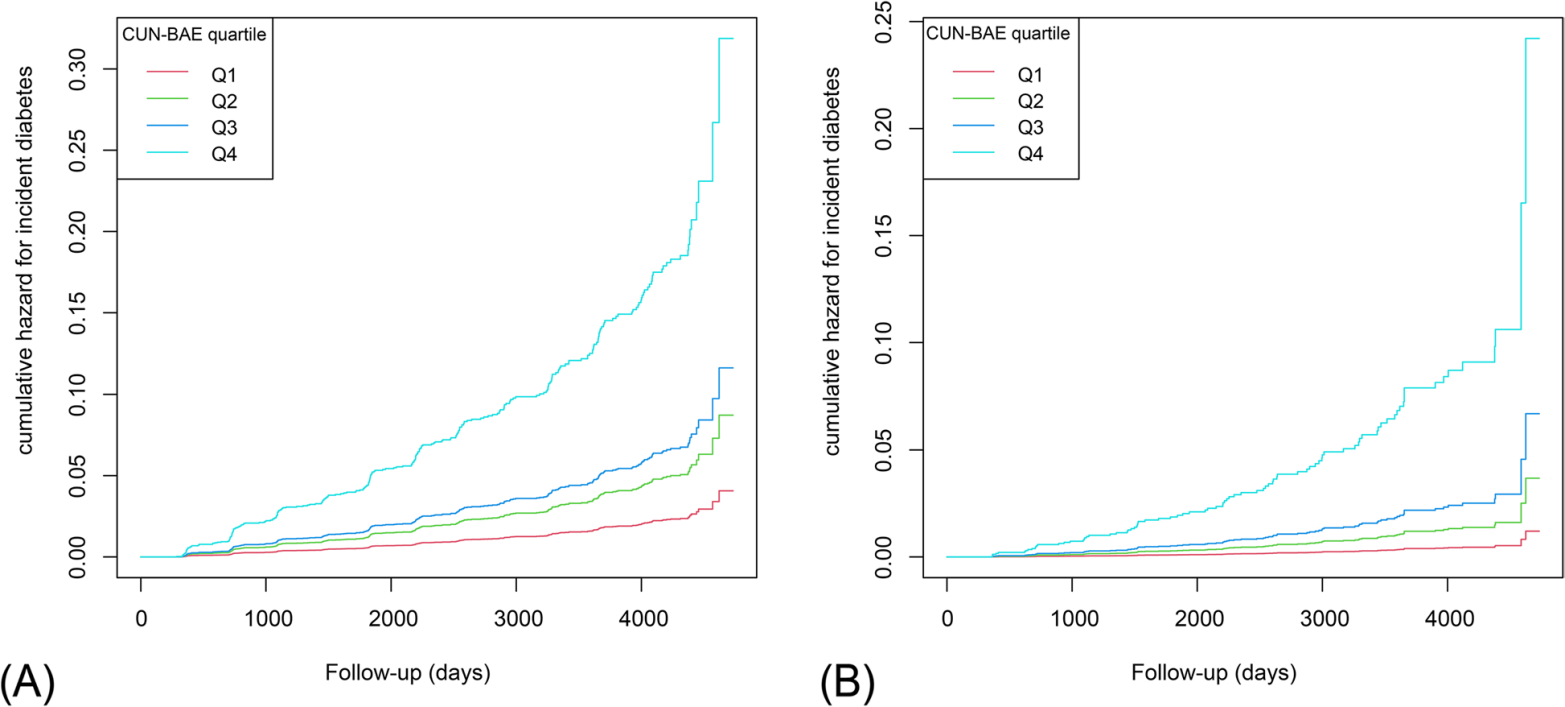
Cumulative incidence of T2DM during follow-up. Notes: The incidence of diabetes increased with ascending CUN-BAE quartiles in both males and females. Both Log-rank *P* < 0.001. **A** Males; **B** Females

Over 5.4 years of follow-up (min-max: 0.5 years–13.0 years) from 2004 to 2015, 373 (2.4%) participants suffered from T2DM in this cohort. As shown in Fig. 3, among different CUN-BAE index quartile groups, the cumulative incidence of diabetes differed considerably (log-rank *P* < 0.001).

### Association between the CUN-BAE and T2DM

As illustrated in Table 2, the unadjusted model of CUN-BAE revealed a significant association with diabetes risk in both sexes (both *P* < 0.001). Significant correction remained after controlling for BMI, age, smoking status, alcohol consumption, exercise habits and fatty liver disease (both *P* < 0.001). The increased baseline CUN-BAE index was still independently linked to an elevated diabetes risk when HDL, TC, TGs, GGT and SBP were also included in Model 3 (both *P* < 0.01). Further, a 2-unit increase in CUN-BAE index was associated with a significantly higher diabetes risk (HR = 1.16 and 1.30 in males and females, respectively, both *P* < 0.01). The top CUN-BAE quartile group remained significantly correlated with incident diabetes after controlling for all variables in Model 2 (*P* < 0.05), while the relationship was moderately attenuated after further adjustment for HDL, TC, TGs, SBP, and GGT in Model 3.

**Table 2:** Association between the baseline CUN-BAE index and incident diabetes among men and women

	Crude		Model 1		Model 2		Model 3	
	HR (95% CI)	*P* value	HR (95% CI)	*P* value	HR (95% CI)	*P* value	HR (95% CI)	*P* value
**Males**								
CUN-BAE (continuous)	1.17 (1.15, 1.20)	< 0.001	1.16 (1.11, 1.21)	< 0.001	1.10 (1.05, 1.15)	< 0.001	1.08 (1.02, 1.14)	0.007
HR for + 2 Units of BF Increase	1.38 (1.39, 1.44)	< 0.001	1.34 (1.22, 1.47)	< 0.001	1.21 (1.09, 1.33)	< 0.001	1.16 (1.04, 1.29)	0.007
CUN-BAE								
Q1	1.0		1.0		1.0		1.0	
Q2	2.15 (1.29, 3.57)	0.003	1.94 (1.16, 3.23)	0.011	1.56 (0.93, 2.61)	0.091	1.38 (0.82, 2.32)	0.228
Q3	2.86 (1.76, 4.65)	< 0.001	2.39 (1.41, 4.03)	< 0.001	1.64 (0.97, 2.80)	0.067	1.31 (0.76, 2.26)	0.328
Q4	7.84 (5.02, 12.25)	< 0.001	4.93 (2.53, 9.61)	< 0.001	2.75 (1.37, 5.52)	0.004	2.04 (1.00, 4.18)	0.051
**Females**								
CUN-BAE (continuous)	1.24 (1.19, 1.28)	< 0.001	1.22 (1.11, 1.33)	< 0.001	1.17 (1.06, 1.28)	< 0.001	1.14 (1.04, 1.25)	< 0.001
HR for + 2 Units of BF Increase	1.53 (1.42, 1.64)	< 0.001	1.48 (1.24,1.77)	< 0.001	1.36 (1.13, 1.63)	< 0.001	1.30 (1.07, 1.57)	< 0.001
CUN-BAE								
Q1	1.0		1.0		1.0		1.0	
Q2	3.05 (0.84, 11.08)	0.090	2.51 (0.69,9.18)	0.163	2.35 (0.64, 8.58)	0.198	2.20 (0.60, 8.06)	0.235
Q3	5.55 (1.63, 18.94)	0.006	3.91 (1.13,13.60)	0.032	3.33 (0.95, 11.62)	0.059	2.97 (0.84,10.45)	0.090
Q4	20.08 (6.29, 64.12)	< 0.001	6.58 (1.81,23.92)	0.004	4.25 (1.15, 15.73)	0.030	3.15 (0.83, 12.00)	0.093

Crude: HR adjusted for none

Model 1: HR adjusted for age, BMI (category)

Model 2: HR adjusted for age, BMI (category), fatty liver disease, smoking status, exercise habits, alcohol consumption

Model 3: HR adjusted for age, BMI (category), fatty liver disease, smoking status, exercise habits, alcohol consumption, HDL, TC, TGs, SBP and GGT

*Abbreviations: HR* hazard ratio, *BF* body fat as captured by the CUN-BAE index

### Stratification analysis and interaction test

**Fig. 4:**
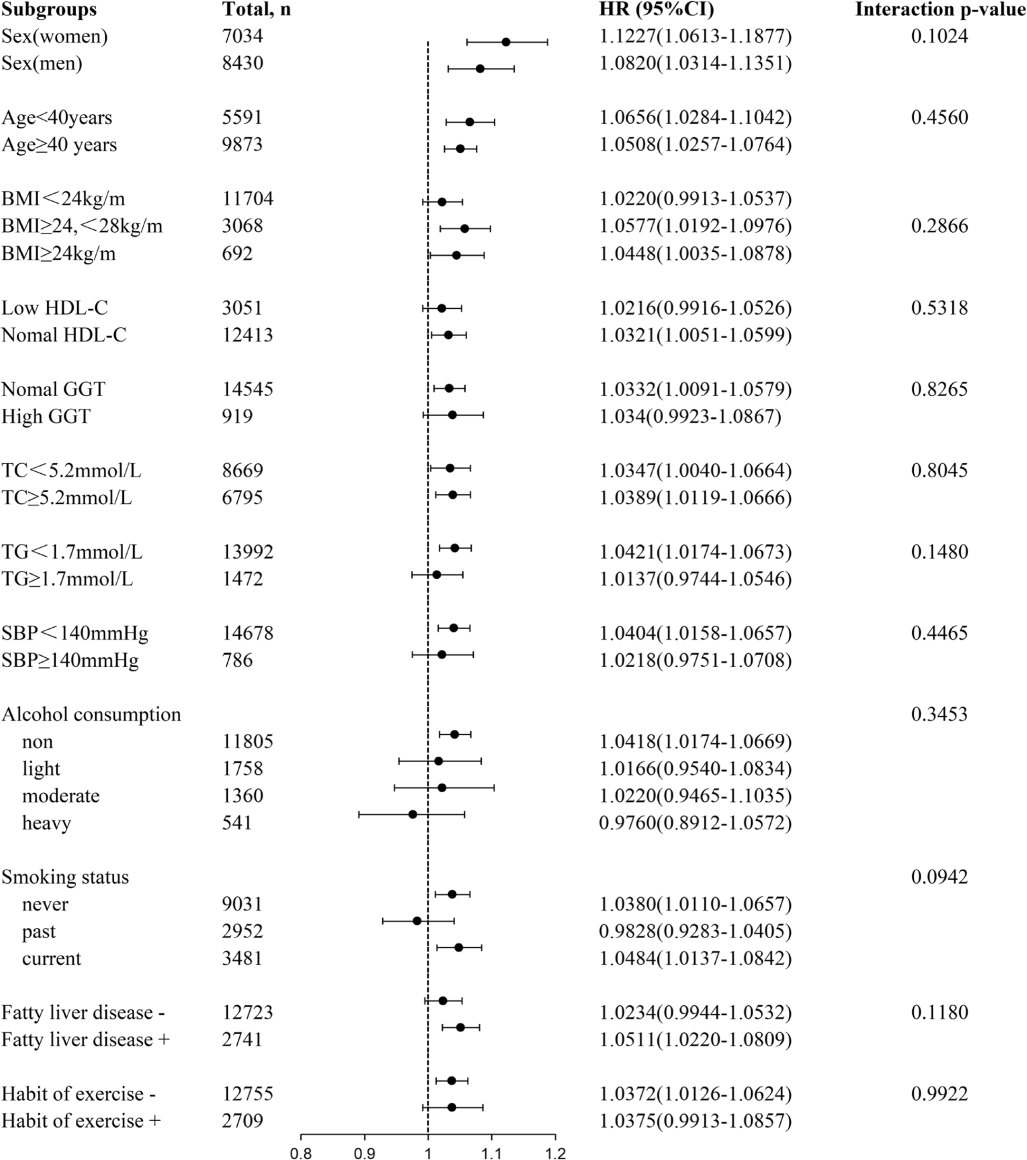
Stratification analysis on the relationship of CUN-BAE index with diabetes occurrence. Notes: HR was calculated using 1 − unit increase of CUN-BAE index. All variables except the stratification variables were included in the stratified analyses of Model 3. HDL < 1.04 mmol/L in men and < 1.29 mmol/L in women was defined as low HDL-C; GGT < 50 U/L in men and < 32 U/L in women was defined as low GGT. Abbreviations: BMI, body mass index; HDL-C, high-density lipoprotein-cholesterol; GGT, gamma-glutamyl transferase; TC, total cholesterol; TGs, triglycerides; SBP, systolic blood pressure

As shown in Fig. 4, subgroup analysis was conducted to identify potential confounding factors affecting the correlation between the baseline CUN-BAE index and type 2 diabetes. All of the covariates used in Model 3 were factored into the model used for the stratified analyses except the variables used for stratification, including sex, age, BMI, smoking status, fatty liver disease, exercise habits, alcohol consumption, HDL, TC, TGs, SBP and GGT. Multiple subgroups analyses of the study found a consistent link between an elevated CUN-BAE index and diabetes incidence (all interaction *P* > 0.05).

### Predictive value of the CUN-BAE index in incident diabetes

**Fig. 5:**
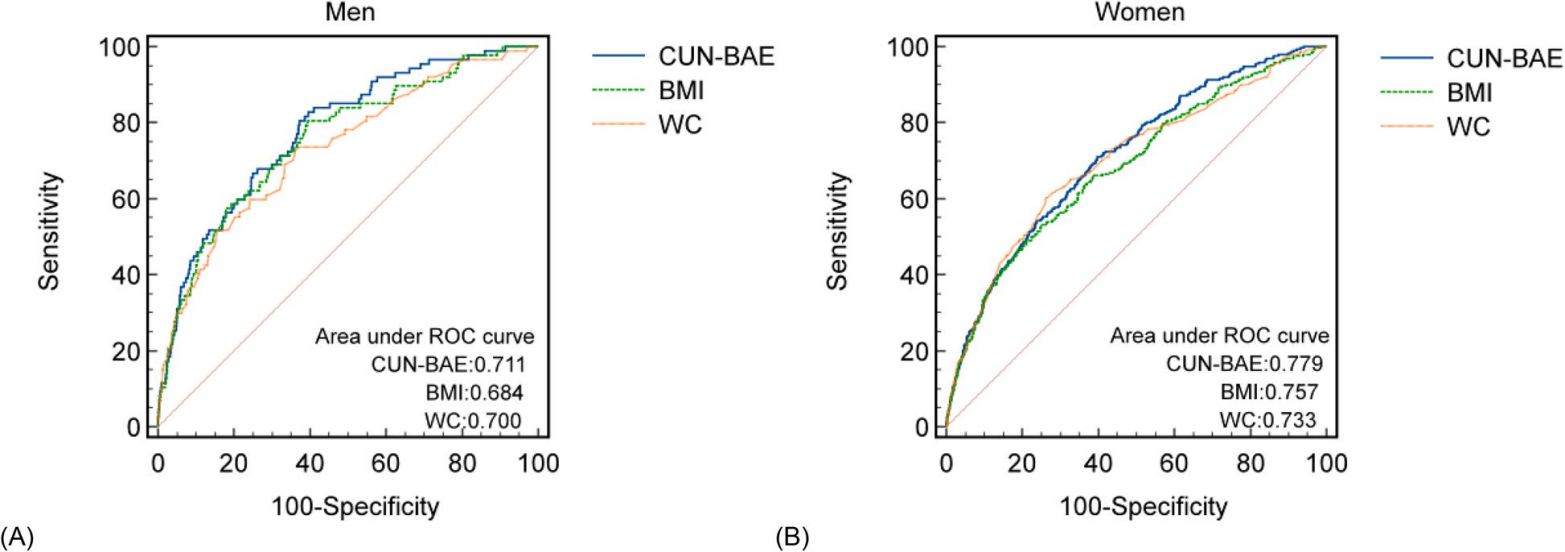
ROC curves of CUN-BAE, BMI and WC for predicting incident T2DM. Notes: **A** Males; **B** Females. Abbreviations: BMI, body mass index; WC, waist circumference; ROC, receiver-operating characteristics

The baseline CUN-BAE index was assessed for its predictive power through the use of an ROC curve (Fig. 5). Among males and females, the areas under the curves (AUCs) of CUN-BAE were 0.711 (95% CI: 0.701–0.721) and 0.779 (95% CI: 0.769–0.788), respectively. In comparison to BMI and WC, the CUN-BAE index demonstrated the largest AUC. In exception to its comparison with WC in male participants, all pairwise AUC comparisons showed significant differences (Table 3).

**Table 3:** Predictive performance of CUN-BAE, BMI and WC for incident diabetes in men and women

Parameters	AUC	95% CI	*P value*	Cut-off points	Specificity	Sensitivity
Men						
CUN-BAE	0.711	0.701–0.721		21.96	0.6030	0.7098
BMI	0.684	0.674–0.694	< 0.001	25.04	0.7893	0.4895
WC	0.700	0.690–0.709	0.2061	84.6	0.7374	0.8465
Women						
CUN-BAE	0.779	0.769–0.788		30.96	0.8046	0.6279
BMI	0.757	0.745–0.767	0.0285	21.23	0.8046	0.6062
WC	0.733	0.722–0.743	0.0040	73.45	0.6303	0.7356

*P value* was calculated by comparing with the AUC of CUN-BAE for prediction of T2DM

*Abbreviations: BMI* body mass index, *WC* waist circumference, *AUC* area under the curve

## Discussion

This population-based study showed an overall increase in the risk of developing type 2 diabetes with increasing CUN-BAE index after potential confounders were taken into account in a Japanese cohort. Compared to BMI and WC, the CUN-BAE index exhibited a better predictive value for diabetes. To our knowledge, this is the first longitudinal study to investigate the relationship between the CUN-BAE index and T2DM, focusing on the Japanese population.

Obesity is a substantial risk factor for T2DM [33]. Prior studies found that the prevalence of obesity assessed by BF% was two to six times higher than that calculated using BMI [29, 34–36]. A possible explanation was that BMI, as a traditional anthropometric measure for general obesity, was unable to differentiate between weight increase caused by muscle and fat, and may thus overlook the slim people with excessive body fat [37, 38]. BF% was proven to be a more precise indicator for obesity identification and obesity-related metabolic diseases, such as T2DM and metabolic syndrome, even in people with normal BMI categories [13, 21]. In recent years, the CUN-BAE index was considered to be an excellent estimation for BF% [28, 39, 40]. In addition, the CUN-BAE index has also been found to be more closely linked to insulin resistance than BMI in males in previous studies [25]. Thus, the CUN-BAE index could be a more meaningful and effective predictor of diabetes.

In the present study, it is remarkable that the CUN-BAE index, as a proxy index for BF%, showed a strong correlation with diabetes incidence. Additionally, our study also revealed that the CUN-BAE was shown to be a more reliable and useful predictor of T2DM incidence than BMI and WC. In a similar manner, diabetes can also be predicted with the CUN-BAE index based on previous studies. A cohort study with 6796 participants from Norway found that CUN-BAE had a closer link with diabetes in both sexes than BMI [28]. In line with our results, CUN-BAE identified more people with metabolic syndrome, diabetes, and hypertension than BMI and other indicators in a European cohort of 12,328 participants [27, 39]. There were several potential reasons for this beneficial relationship. First, the accumulation of fat enhances the release of free fatty acids and results in increased lipid accumulation [19]. This induces insulin resistance by activating the diacylglycerol-protein kinase C pathway [20]. Second, excess body fat results in the dysregulation of a wide range of adipokines including classic hormones such as leptin, which may contribute to diabetes via the alteration of glucose metabolism, lipid metabolism and inflammation [17, 18, 41]. Third, individuals who are genetically susceptible to diabetes show a greater risk of obesity because there is a tendency toward insulin resistance in their skeletal muscle and pancreatic islet β-cells [42, 43]. However, another cross-sectional study including 69,388 Chinese participants aged ≥60 years showed that BMI and CUN-BAE were less reliable predictors of male health than WC [43]. This discrepancy may be caused by the diverse ages of the study population, but it could also be a result of the different study methodologies and designs.

## Comparisons with other studies and what does the current work add to the existing knowledge

CUN-BAE is a body fat prediction equation that has been proven to be linked to hypertension, cardiovascular events and cardiometabolic risk factors in prior studies [25, 27–29]; however, these relationships have only been explored in Caucasian populations. In addition, the predictive potential of CUN-BAE for incident diabetes has not yet been compared with BMI and WC. We first revealed the level of CUN-BAE in a Japanese population. Compared with other studies, we observed that the level of the CUN-BAE index varies across different ethnic groups. The present study showed that the CUN-BAE was 24.96 ± 6.52 in this Japanese population (20.9 ± 4.7 in males and 29.8 ± 4.9 in females), which is similar to findings in a Chinese population [44, 45]. The mean level of CUN-BAE was 29.8 ± 7.8 in a Spanish population (25.4 ± 6.6 in males and 33.5 + 6.7 in females) [40]. These findings are similar to the results of a study by Davila-Batista et al. [46] but higher than the results of a study in South Africa (27.28 ± 8.28) [47]. It is possible that this discrepancy is due to differences in body composition, body size, and body fat distribution among various ethnic groups. Asian individuals tend to be small and have a low BMI, while Caucasian individuals are more likely to be large and muscular with a high BMI. Furthermore, we demonstrate an independent association of CUN-BAE with diabetes and its clinical usefulness for diabetes prediction in an Asian population, providing new evidence for the application and promotion of CUN-BAE in Asian ethnic groups. Moreover, this is the first experiment to find that CUN-BAE exceeds BMI and WC in diabetes prediction, which is beneficial for the accurate and early identification of patients at risk of diabetes and might have potential reference value for the adjustment of treatment strategies.

## Study strengths and limitations

There are several advantages in the present study. First, this study is the first longitudinal study that investigated the link between CUN-BAE and diabetes in a Japanese population. Second, we made comparisons with traditional anthropometric indicators, including BMI and WC, to further evaluate the clinical value of the CUN-BAE index. Third, subgroup analysis was conducted to ensure that CUN-BAE and T2DM were associated in a stable manner among different participants.

Nevertheless, several limitations are inherent in the study. First, the findings might be difficult to generalize to other ethnicities, as the present study only considers the Japanese population. Second, HbA1c, FPG, or patient self-reported data were primarily used for diabetes diagnosis, rather than oral glucose tolerance tests (OGTTs), in this study, which may result in an underestimation of the prevalence of diabetes. Third, the CUN-BAE index was evaluated at baseline. Thus, it did not consider the dynamic changes over time, which may profoundly affect the incidence of diabetes. Finally, we could not fully adjust the confounding factors in the original study that could affect the results.

## Conclusions

In conclusion, diabetes incidence is significantly correlated with increased adiposity assessed by the CUN-BAE index. The CUN-BAE index is more effective in predicting T2DM than both BMI and WC. It could be employed as a prominent indicator for the early detection and prediction of a high risk of T2DM in clinical practice.

## Data Availability

Data for the study can be obtained from the “Dryad” database, which is publicly accessible. (https://datadryad.org/stash/data/set/doi:10.5061/dryad.8q0p192).

## Abbreviations

CUN-BAE: The Clínica Universidad de Navarra-Body Adiposity Estimator index
BMI: Body mass index
WC: Waist circumference
SBP: Systolic blood pressure
DBP: Diastolic blood pressure
TC: Total cholesterol
TG: Triglycerides
HDL-C: High-density lipoprotein-cholesterol
FBG: Fasting blood glucose
HbA1c: Glycosylated haemoglobin A1c
ALT: Alanine aminotransferase
AST: Aspartate aminotransferase
GGT: Gamma-glutamyl transferase
ROC: Receiver-operating characteristics
AUC: Area under the curve
SD: Standard deviation
HR: Hazard ratio
CI: Confidence interval
T2DM: Type 2 diabetes mellitus
NAGALA: NAfld in Gifu Area, Longitudinal Analysis
